# Association study of apelin-APJ system genetic polymorphisms with incident metabolic syndrome in a Chinese population: a case-control study

**DOI:** 10.18632/oncotarget.24111

**Published:** 2019-06-04

**Authors:** Meijin Zhang, Feng Peng, Liming Lin, Mingzhong Yu, Chengyuan Huang, Dan Hu, Qinghui Guo, Changsheng Xu, Jinxiu Lin

**Affiliations:** ^1^ The First Clinical Medical College of Fujian Medical University, Fuzhou, Fujian, China; ^2^ Department of Cardiology, The First Affiliated Hospital, Fujian Medical University, Fuzhou, Fujian, China; ^3^ Department of Cardiology, The Affiliated Hospital of Putian University, Putian, Fujian, China; ^4^ Department of Cadre Ward, The First Affiliated Hospital, Fujian Medical University, Fuzhou, Fujian, China; ^5^ Department of Neurology, Zhangzhou Affiliated Hospital of Fujian Medical University, Zhangzhou, Fujian, China; ^6^ Department of Pathology, Fujian Provincial Cancer Hospital, The Affiliated Hospital of Fujian Medical University, Fuzhou, Fujian, China; ^7^ Fujian Provincial Institute of Hypertension, Fuzhou, Fujian, China

**Keywords:** apelin, APJ, metabolic syndrome, single nucleotide polymorphism

## Abstract

**Objectives:** Apelin-APJ system has been implicated in the regulation of metabolic homeostasis. This study aimed to explore the genetic predisposition of the apelin-APJ system to metabolic syndrome.

**Materials And Methods:** 1005 subjects were enrolled, including 448 metabolic syndrome patients and 557 controls. Seven single nucleotide polymorphisms, including rs909656, rs5975126, and rs3115757 of the apelin gene and rs7119375, rs10501367, rs9943582 and rs11544374 of the APJ gene, were genotyped.

**Results:** For males, apelin-36 were higher in metabolic syndrome subjects compared with controls (*p* < 0.05). Apelin-36 were significantly lower in those with TT genotype of rs10501367 than those with CC and CT genotypes (*p* < 0.05), and fasting plasma glucose were higher in T allele carriers of rs10501367 and A allele carriers of rs7119375 compared with non-carriers (both *p* < 0.05). A significant difference in genotype distribution between diabetes mellitus patients and controls existed for both rs10501367 and rs7119375 (both *p* < 0.05). However, the association between apelin-APJ system genetic polymorphisms and metabolic syndrome was nonsignificant.

For females, apelin-36 were higher in metabolic syndrome subjects compared with controls (*p* < 0.05). The association between apelin-APJ system genetic polymorphisms and apelin-36, fasting plasma glucose and diabetes mellitus was nonsignificant. However, carrying A allele in rs7119375 was associated with lower metabolic syndrome risk compared with non-carriers of A allele (odds ratio: 0.646, 95% confidence interval: 0.420–0.994, *p* = 0.043).

**Conclusions:** The current findings revealed a gender-specific association of apelin-APJ system genetic polymorphisms with metabolic syndrome and glucose homeostasis disorders in a Han Chinese population.

## INTRODUCTION

A growing body of evidence indicating a critical role for apelin implicated in the regulation of metabolic homeostasis by activating the APJ receptor, a G-protein-coupled 7-transmembrane receptor, has accumulated. Plasma apelin levels were significantly higher in obese patients compared to control individuals [1–2], while the elevated plasma apelin levels were reversed by weight loss induced by a hypo-caloric diet [3]. In animal models, apelin-transgenic mice were resistant to diet-induced obesity via stimulating energy expenditure [4] and administration of apelin-13 (a truncated apelin peptide with 13-amino acid) decreased body adiposity in diet-induced obese mice [5]. Additionally, plasma apelin concentrations were also increased in diabetic patients [6–7] wherein apelin was involved in the maintenance of insulin sensitivity [8] through enhanced glucose utilization [9]. For lipid metabolism, inhibition of adipogenesis and lipolysis by apelin was documented [10] and a significant association between apelin and triglycerides (TG) was revealed in morbidly obese patients with type 2 diabetes [6]. Apelin was also identified as a vasodilator with blood pressure-lowering properties via a nitric oxide-dependent mechanism [11–12]. Furthermore, mounting evidence has indicated a predictive role for single nucleotide polymorphisms (SNPs) in the apelin-APJ system for incident hypertension [13], obesity phenotype and insulin resistance [14]. Taken together, the apelin-APJ system and its genetic polymorphisms have been shown to be correlated with components of metabolic syndrome (MetS), which is characterized as a constellation of metabolic disturbances. However, the role of the apelin-APJ pathway in the pathogenesis of MetS is not fully elucidated yet.

We were aimed at exploring the genetic predisposition of the apelin-APJ system to MetS. To address this issue, a hospital-based case-control trial was conducted in a Han Chinese population with a total of 1005 inpatients in The First Affiliated Hospital of Fujian Medical University. Previous studies have also demonstrated that the renin-angiotensin-aldosterone system (RAAS) and sympathetic nervous system (SNS) played a crucial role in the pathophysiology of MetS [15–16]. Thus, RAAS and SNS associated parameters were taken as traits in the genetic association analysis in this work.

## RESULTS

### Association between apelin-APJ system genetic polymorphisms and MetS related parameters

### Basic characteristics, plasma apelin-36 levels, SNS and RAAS related parameters

A total of 1005 subjects were recruited in this study, comprising 448 MetS patients and 557 controls. Anthropometric indices and clinical laboratory biomarkers of all the participants are summarized in Table 1. Compared with controls, MetS patients were older and had significantly higher levels of waist circumference (WC), TG, fasting plasma glucose (FPG), angiotensin II (Ang II), angiotensin-converting enzyme 2 (ACE2), and apelin-36, but lower levels of high density lipoprotein cholesterol (HDL-c) for both sexes (all *p* < 0.05). Meanwhile, the levels of diastolic blood pressure (DBP) were significantly higher in MetS patients than in controls for males. None of the other parameters exhibited a significant difference between MetS patients and controls for both genders.

**Table 1:** Gender-stratified comparison of anthropometric indices, clinical laboratory biomarkers, plasma apelin-36 levels, SNS and RAAS related parameters between MetS patients and controls

	**Male**		**Female**	
	**Control (*n* = 309)**	**MetS (*n* = 272)**	**Control (*n* = 248)**	**MetS (*n* = 176)**
age (years)	59.25 ± 13.72	61.78 ± 11.81^*^	58.16 ± 14.18	65.56 ± 10.26^*^
WC (cm)	82.9 ± 7.6	93.97 ± 8.02^*^	80.28 ± 8.85	90.68 ± 8.46^*^
TC (mmol/L)	4.1209 ± 0.93	4.0722 ± 1.02	4.4316 ± 0.9	4.4102 ± 1.03
TG (mmol/L)	1.107 ± 0.59	1.9745 ± 1.4^*^	1.1645 ± 0.55	1.877 ± 0.9^*^
HDL-c (mmol/L)	1.1382 ± 0.28	0.9042 ± 0.23^*^	1.2842 ± 0.28	1.0387 ± 0.25^*^
LDL-c (mmol/L)	2.7086 ± 0.85	2.5843 ± 0.95	2.8227 ± 0.84	2.7439 ± 0.92
FPG (mmol/L)	4.748 ± 0.93	5.624 ± 1.68^*^	4.835 ± 0.8	6.051 ± 2.06^*^
SBP (mmHg)	170.42 ± 21.16	174.49 ± 23.28	177.62 ± 22.3	180.45 ± 22.21
DBP (mmHg)	97.51 ± 17.12	101.8 ± 16.9^*^	99.12 ± 14.77	98.75 ± 16.84
rennin (clinostatism) (ng/ml^*^h)	2.0325 ± 2.41	2.1359 ± 3	1.9212 ± 2.61	1.7702 ± 3.06
Ang I (clinostatism) (ng/dl)	2.4899 ± 3.13	2.5927 ± 3.72	2.2707 ± 3.14	2.2945 ± 3.91
aldosterone (clinostatism) (ng/dl)	12.6971 ± 3.5	13.281 ± 3.34	12.2551 ± 3.07	11.4142 ± 3.48
epinephrine (ng/l)	158.78 ± 38.89	156.66 ± 33.54	154.61 ± 28.72	162.86 ± 34.76
norepinephrine (ng/l)	342.81 ± 114.42	392.43 ± 326.21	344.61 ± 73.18	381.61 ± 141.9
dopamine (ng/l)	127.44 ± 36.73	124.89 ± 21.42	124.47 ± 23.89	131.19 ± 25.76
Ang II (ng/l)	91.78 ± 26.45	109.94 ± 30.74^*^	103.78 ± 34.72	116.3 ± 33.77^*^
ACE2 (ng/l)	97.72 ± 27.72	114.59 ± 27.46^*^	100.55 ± 31.19	114.25 ± 27.62^*^
apelin-36 (ng/l)	1050.39 ± 340.25	1264.84 ± 362.29^*^	946.59 ± 326.12	1295.34 ± 282.24^*^

Qualitative data were shown as the mean and standard deviation (mean ± SD) and quantitative data were presented as counts and percentages. SNS, sympathetic nervous system; RAAS, renin-angiotensin-aldosterone system; MetS, metabolic syndrome; WC, waist circumference; TC, total cholesterol; TG, triglycerides; HDL-c, high-density lipoprotein cholesterol; LDL-c, low-density lipoprotein cholesterol; FPG, fasting plasma glucose; SBP, systolic blood pressure; DBP, diastolic blood pressure; Ang Ι, angiotensin Ι; Ang II, angiotensin II; ACE2, angiotensin-converting enzyme 2. ^*^P value < 0.05 compared with controls.

### Association between apelin-APJ system genetic polymorphisms with MetS individual components, plasma apelin-36 levels and RAAS related parameters

**Figure 1:**
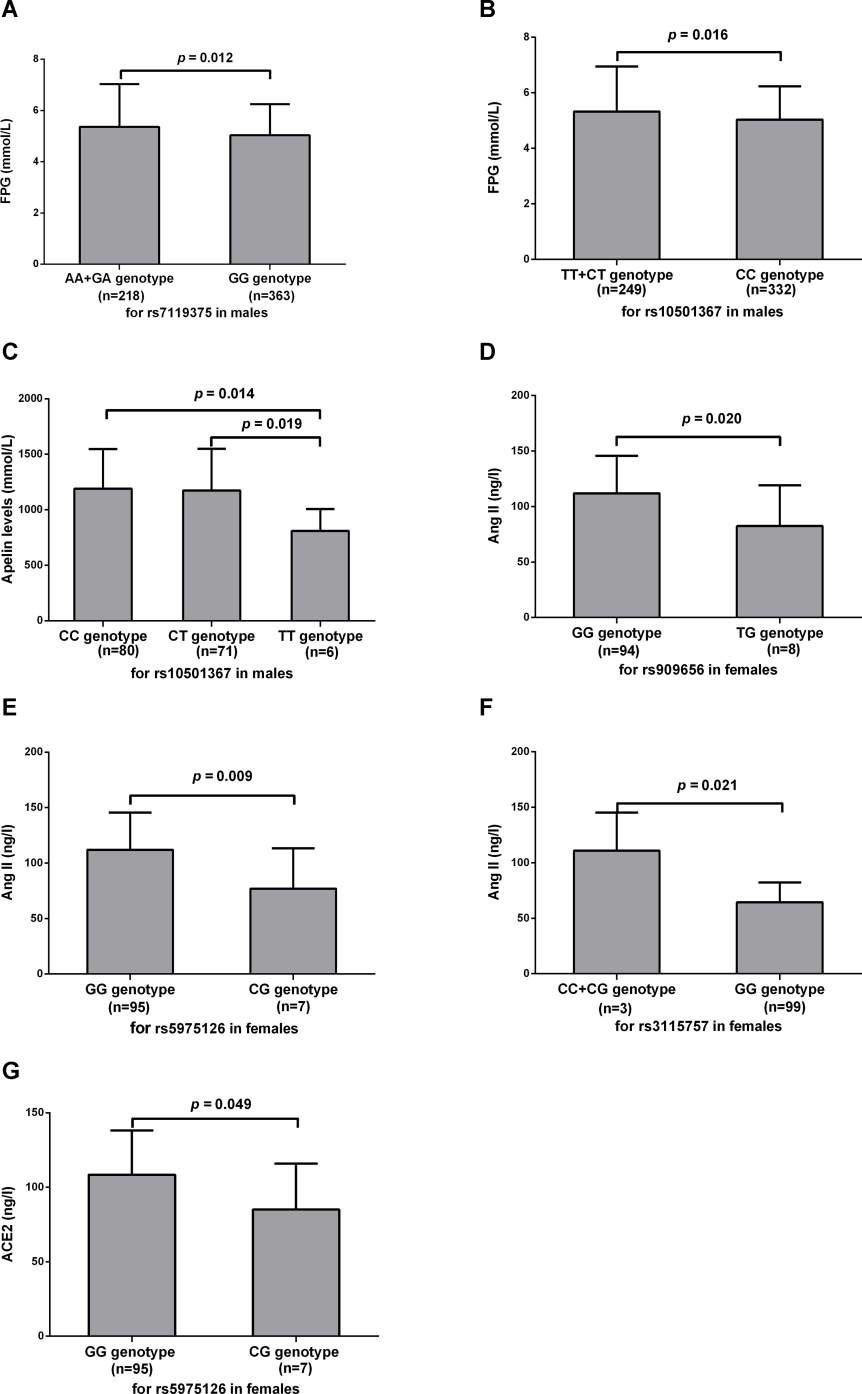
Association between apelin-APJ system with MetS individual components and RAAS related parameters. Levels of FPG (**A**–**B**), apelin-36 (**C**), Ang II (**D**–**F**) and ACE2 (**G**) were compared between different genotype groups. Values were represented as the mean and standard deviation. MetS, metabolic syndrome; FPG, fasting plasma glucose; Ang II, angiotensin II; ACE2, angiotensin-converting enzyme 2.

As displayed in Figure 1 and the Supplementary Table 5, for males, FPG levels differed significantly across different genotypes of rs7119375 (*p* = 0.006), with significantly higher values in A allele carriers (AA + GA genotype) compared with G allele homozygous carriers (GG genotype) (5.36 ± 1.66 vs. 5.04 ± 1.21 mmol/L, *p* = 0.012). Similar phenomena were observed across different genotypes of rs10501367 (*p* = 0.014), with higher levels of FPG in T allele carriers (TT + CT genotype) relative to C allele homozygous carriers (CC genotype) (5.33 ± 1.63 vs. 5.03 ± 1.20mmol/L, *p* = 0.016). For females, however, there existed no significant difference in FPG levels across different genotypes of rs7119375 and rs10501367. In terms of other MetS components including WC, DBP, TG, HDL-c and systolic blood pressure(SBP), there existed no significant difference across different genotypes for all the examined SNPs.

For males, there was a borderline difference in plasma apelin-36 levels among three genotypes of rs10501367 (1189.49 ± 358.96, 1173.82 ± 375.36 and 808.98 ± 197.41 ng/l for CC, CT and TT, respectively, *p* = 0.048). Apelin-36 levels in patients with the TT genotype were significantly lower than those with the CC and CT genotypes (*p* = 0.014 and 0.019, respectively), whereas apelin-36 levels between CC and CT genotype were similar (*p* = 0.791). No difference was observed across allele or genotype groups for rs10501367 in females and for rs909656, rs5975126, rs3115757, rs7119375, rs9943582 or rs11544374 in both sexes.

For females, levels of Ang II were significantly higher in patients with the GG genotype of rs909656 compared with the TG genotype (111.87 ± 33.686 vs. 82.32 ± 36.925 ng/l, *p* = 0.020) and were significantly higher in patients with the GG genotype of rs5975126 compared with the CG genotype (111.95 ± 33.517 vs. 76.93 ± 36.326 ng/l, *p* = 0.009). A significant difference in Ang II levels was also seen for rs3115757 in females, with elevation of Ang II in C allele carriers (CC + CG genotype) relative to G allele homozygous carriers (GG genotype) (110.92 ± 34.190 vs. 64.35 ± 17.887 ng/l, *p* = 0.021). A difference in Ang II was not statistically significant for rs909656, rs5975126 and rs3115757 in males, and rs7119375, rs10501367, rs9943582 or rs11544374 for both genders. A marginal significance was identified between the GG genotype and CG genotype of rs5975126 for females regarding ACE2 (108.46 ± 29.746 vs. 85.18 ± 30.792 ng/l, *p* = 0.049). No statistical significance was observed between groups for rs5975126 in males, and rs909656, rs3115757, rs7119375, rs10501367, rs9943582 or rs11544374 in both sexes.

### Association between apelin-APJ system genetic polymorphisms and MetS

### Hardy-Weinberg equilibrium test for the examined SNPs

Given that the apelin gene (*APLN*) is located on the X chromosome, the Hardy-Weinberg equilibrium test of genetic polymorphisms in *APLN* was only conducted in females. The distribution of the examined SNPs conformed to Hardy-Weinberg equilibrium with a significance level of 5% (Supplementary Table 4).

### Association between apelin-APJ system genetic polymorphisms and MetS

As depicted in Table 2, for females, no significant difference in the genotype distribution of rs7119375 existed between MetS patients and controls, however, the allele distribution of rs7119375 differed between groups, with the A allele slightly but significantly less common in MetS patients than in controls (84.4% vs. 79.5%, *p* = 0.0495). After adjustment for age, the A allele carriers (AA+GA genotype) had a reduced risk of incident MetS compared to those with G allele homozygous carriers (GG genotype) (odds ratio (OR): 0.646, 95% confidence interval (95% CI): 0.420-0.994, *p* = 0.043). For males, however, the rs7119375 variant was not significantly associated with MetS incidence. In terms of rs909656, rs5975126, rs3115757, rs10501367, rs9943582 and rs11544374, there was no significant difference in the genotype or allele distribution between MetS patients and controls for both genders.

**Table 2:** Gender-stratified association between apelin-APJ system genetic polymorphisms and MetS

**SNP**		**Male**			**Female**	
**Control (*n* = 309)**	**MetS (*n* = 272)**	***p* value**	**Control (*n* = 248)**	**MetS (*n* = 176)**	***p* value**	
apelin: rs909656						
GG	^-^a	-		229 (92.3%)	161 (91.5%)	0.489
GT	-	-		19 (7.7%)	14 (8.0%)	
TT	-	-		0 (0%)	1 (0.6%)	
G	301(97.4%)	266 (97.8%)	0.764	477 (96.2%)	336 (95.5%)	0.606
T	8 (2.6%)	6 (2.2%)		19 (3.8%)	16 (4.5%)	
T vs. ^G^b	Reference	0.888 (0.301-2.615)	0.780^c^			
TT+TG vs. ^G^Gb				Reference	0.969 (0.465-2.019)	0.969^d^
rs5975126						
GG	-	-		230 (92.7%)	162 (92.0%)	0.789
GC	-	-		18 (7.3%)	14 (8.0%)	
CC	-	-		0 (0%)	0 (0%)	
G	302 (97.7%)	267 (98.2%)	0.718	478 (96.4%)	338 (96.0%)	0.793
C	7 (2.3%)	5 (1.8%)		18 (3.6%)	14 (4.0%)	
C vs. ^G^b	Reference	0.866 (0.269-2.791)	0.728^c^			
CC+CG vs. G^G^b				Reference	0.954 (0.448-2.032)	0.938^d^
rs3115757						
GG	-	-		18 (7.3%)	7 (4.0%)	0.264
GC	-	-		95 (38.3%)	63 (35.8%)	
CC	-	-		135 (54.4%)	106 (60.2%)	
G	78 (25.2%)	55 (20.2%)	0.151	365 (73.6%)	275 (78.1%)	0.130
C	231 (74.8%)	217 (79.8%)		131 (26.4%)	77 (21.9%)	
C vs. G ^b^	Reference	0.758 (0.511-1.127)	0.16^2^c			
CC+CG vs. G^G^b				Reference	1.671 (0.663-4.214)	0.296^d^
APJ: rs7119375						
GG	191 (61.8%)	172 (63.2%)	0.886	153 (61.7%)	124 (70.5%)	0.131
GA	102 (33.0%)	88 (32.4%)		86 (34.7%)	49 (27.8%)	
AA	16 (5.2%)	12 (4.4%)		9 (3.6%)	3 (1.7%)	
G	484 (78.3%)	432 (79.4%)	0.649	392 (79.5%)	297 (84.4%)	0.0495
A	134 (21.7%)	112 (20.6%)		104 (20.5%)	55 (15.6%)	
AA+GA vs. GG^b^	Reference	0.986 (0.700-1.387)	0.850^d^	Reference	0.646 (0.420-0.994)	0.043^d^
rs10501367						
CC	178 (57.6%)	154 (56.6%)	0.868	137 (55.2%)	112 (63.6%)	0.161
CT	114 (36.9%)	105 (38.6%)		100 (40.3%)	60 (34.1%)	
TT	17 (5.5%)	13 (4.8%)		11 (4.4%)	4 (2.3%)	
C	470 (76.05%)	413 (75.9%)	0.958	374 (75.4%)	284 (80.7%)	0.069
T	148 (23.95%)	131 (24.1%)		122 (24.6%)	68 (19.3%)	
TT+CT vs. CC^b^	Reference	1.080 (0.774-1.507)	0.712^d^	Reference	0.752 (0.497-1.138)	0.173^d^
rs9943582						
CC	198 (64.1%)	176 (64.7%)	0.679	166 (67.0%)	110 (62.5%)	0.631
CT	95 (30.7%)	86 (31.6%)	70 (28.2%)	57 (32.4%)		
TT	16 (5.2%)	10 (3.7%)	12 (4.8%)	9 (5.1%)		
C	491 (79.5%)	438 (80.5%)	0.651	402 (81.1%)	277 (78.7%)	0.398
T	127 (20.5%)	106 (19.5%)	94 (18.9%)	75 (21.3%)		
TT+CT vs. CC^b^	Reference	0.973 (0.690-1.372)	0.888^d^	Reference	1.229 (0.805-1.876)	0.298^d^
rs11544374						
GG	218 (70.6%)	183 (67.3%)	0.538	166 (66.9%)	130 (73.9%)	0.118
GA	81 (26.2%)	82 (30.1%)	75 (30.2%)	45 (25.6%)		
AA	10 (3.2%)	7 (2.6%)	7 (2.8%)	1 (0.6%)		
G	517 (83.7%)	448 (82.4%)	0.554	407 (82.0%)	305 (86.6%)	0.073
A	101 (16.3%)	96 (17.6%)	89 (18.0%)	47 (13.4%)		
AA+GA vs. GG^b^	Reference	1.221(0.854-1.747)	0.283^d^	Reference	0.648 (0.414-1.015)	0.051^d^

MetS, metabolic syndrome; SNP, single nucleotide polymorphism. ^a^Because *APLN* is on the X chromosome, genotype information in males is unavailable. ^b^Values were shown as odds ratios (95% confidence intervals). ^c^ *p* values were forallelic model of inheritance after adjusting for age using binary logistic regression analysis. ^d^ *p* values were for dominant model of inheritance after adjusting for age using binary logistic regression analysis.

### Association between haplotypes in the apelin-APJ system and metS

To investigate the combinational effects of different SNPs on MetS incidence, a haplotype analysis was conducted. Seeing that *APLN* and the APJ gene (*APLNR*) are located on different chromosomes, haplotype analysis was performed separately. Linkage disequilibrium maps for *APLN* SNPs and *APLNR* SNPs are shown in Supplementary Figure 1, and the sex-specific haplotype distribution of the *APLN* and *APLNR* with a frequency > 0.05 is summarized in Table 3.

**Table 3:** Gender-stratified haplotype analysis for apelin-APJ system polymorphisms between MetS patients and controls

**Haplotype**	**Male (*n* = 581)**			**Female (*n* = 424)**		
	**Control**	**MetS**	***p* value**	**Control**	**MetS**	***p* value**
*APL*^N^a						
G-C-G	72.17%	77.57%	0.1348	70.87%	74.83%	**0.2013**
G-G-G	25.24%	20.22%	0.1505	25.30%	20.63%	**0.1117**
*APLNR*^b^						
C-G	75.3%	73.8%	0.557	74.8%	79.5%	0.10
T-A	21.0%	18.5%	0.2931	20.3%	14.5%	0.02

MetS, metabolic syndrome; *APLN*, the apelin gene; *APLNR*, the APJ gene. ^a^ Haplotypes for *APLN* were in order of rs5975126, rs3115757 and rs909656. ^b^ Haplotypes for *APLNR* were in order of rs10501367 and rs7119375.

For *APLN*, Haploview software was used to estimate two haplotypes formed by alleles in order of rs5975126, rs3115757 and rs909656, and for *APLNR*, two haplotypes were defined according to the order of rs10501367 and rs7119375. In females, the T-A haplotype in *APLNR* was over-represented in the controls (20.3% vs. 14.5% in MetS), and a significant difference for the haplotype distribution was observed between MetS patients and controls (*p* = 0.0281). No significant difference in haplotype frequencies for males was detected.

### Association between apelin-APJ system genetic polymorphisms and incident diabetes mellitus (DM)

### Association between apelin-APJ system genetic polymorphisms and DM

The population was sorted as DM patients and controls, and the genotype distribution of rs909656, rs5975126, rs3115757, rs7119375 and rs10501367, which were correlated with FPG and RAAS, was analysed between groups (Table 4). For males, a significant difference in the genotype distribution between DM patients and controls existed for both rs7119375 and rs10501367 after controlling for age, WC, smoking and drinking status (*p* = 0.046 and *p* = 0.020, respectively). In contrast, no significant difference was observed for females and rs909656, rs5975126 or rs3115757.

**Table 4:** Gender-stratified association between apelin-APJ system genetic polymorphisms with incident DM

**SNP**	**Gender**	***n***	***p* value**
rs909656	Male	581	0.404
(TT vs. TG vs. GG)	Female	424	0.667
rs5975126	Male	581	0.591
(CC vs. CG vs. GG)	Female	424	0.700
rs3115757	Male	581	0.369
(CC vs. CG vs.GG)	Female	424	0.877
rs7119375	Male	581	0.046
(AA vs. GA vs. GG)	Female	424	0.633
rs10501367	Male	581	0.020
(TT vs. CT vs. CC)	Female	424	0.792

DM, diabetes mellitus; SNP, single nucleotide polymorphism.

### Association between rs10501367 polymorphisms and DM in males

Our data showed that rs10501367 was associated significantly with apelin-36 levels, FPG levels and DM risk in males, thus single-locus analysis of rs10501367 was further conducted in males between DM patients and controls (Table 5). After adjustment for age, WC, smoking and drinking status, the T allele homozygous carriers (TT genotype) was associated with an enhanced risk of incident DM compared to those with C allele carriers (CC + CT genotype) (OR: 2.256, 95% CI: 1.022-4.979, *p* = 0.044). No significant difference was detected for the allele distribution or genotype distribution under a dominant genetic model.

**Table 5:** Association between rs10501367 polymorphisms and DM in males

	**Control (*n* = 393)**	**DM (*n* = 188)**	***p* value**	**OR (95% CI)**
C	610 (77.6%)	273 (72.6%)	0.062	0.765 (0.577~1.014)
T	176 (22.4%)	103 (27.4%)		
TT + CT	159 (40.5%)	90 (47.9%)	0.058	1.430 (0.988–2.071)
CC	234 (59.5%)	98 (52.1%)		
TT	17 (4.3%)	13 (6.9%)	0.044	2.256 (1.022–4.979)
CC + CT	376 (95.7%)	175 (93.1%)		

DM, diabetes mellitus; OR, odds ratio; 95%CI, 95% confidence interval.

## DISCUSSION

In this work, we sought to investigate the genetic role of the apelin-APJ system in MetS. As a constellation of metabolic disturbances including hyperglycaemia, dyslipidaemia, hypertension and obesity, MetS predisposes patients to DM type 2 [17], coronary heart disease [18–19] and other diseases. The multiple components of MetS coexist more often than by chance alone. Mounting evidence has highlighted the effect of genetics, central obesity and insulin resistance in the development of MetS. However, the pathogenesis for MetS is still inadequately understood. As aforementioned, the apelin-APJ pathway is implicated in the regulation of multiple metabolic disturbances, whereas data on the interaction between the apelin-APJ pathway and MetS are still sparse, and no evidence on the susceptibility of common genetic polymorphisms in the apelin-APJ system to MetS has been gathered [20]. To our knowledge, our findings suggested for the first time a gender-specific predisposition of apelin-APJ system genetic polymorphisms to MetS incidence and its components in a Han Chinese population, which may be tied to the alteration of plasma levels of apelin-36.

The current study confirmed significantly higher apelin-36 levels in MetS patients than controls in both genders. The same result was found for males by Angelova P et al. [21–22]. Karbek B et al. also documented higher levels of apelin-12 and apelin-36 in MetS than age-matched controls, and revealed a positive relationship between apelin-12 and apelin-36 levels with insulin resistance, which is the core of MetS [23]. These data suggest that apelin has important clinical implications for MetS.

It is of great interest to assess the genetic effect of the apelin receptor as it is responsible for signal transduction. In the genetic association analysis, the TT genotype of rs10501367 in the *APLNR* gene was associated with lower apelin-36 levels than CC and CT genotypes in males. To date, few studies have investigated the association between APJ genetic polymorphisms and apelin levels in a Chinese population. In contrast to our results, the G allele carriers of the APJ polymorphism rs11544374 were associated with lower apelin levels in a Greek population [24]. Another APJ polymorphism A445C presented no difference in apelin levels in a Korean population [25]. SNP function prediction for rs10501367 using the web tool FastSNP showed that rs10501367 influences gene transcription through binding to a transcription factor. However, the molecular mechanism of how the APJ gene modulates apelin expression has remained unclear. Furthermore, the small sample size of this single marker analysis made our study underpowered. Therefore, a larger population is desired and studies using a luciferase activity assay are needed to elucidate functional relations.

We found that Ang II and ACE2 levels were significantly higher in MetS compared with controls regardless of gender. Furthermore, for females, a significant difference of Ang II was noted across different genotypes of rs5975126, rs3115757 and rs909656, and ACE2 levels were statistically higher in GG genotype of rs5975126. Many studies have revealed the high sequence identity of apelin and APJ to Ang II [11] and the angiotensin II type 1 receptor [26], respectively. Apelin can be specifically degraded by ACE2 [27], a close homologue to angiotensin I converting enzyme. Additionally, the tissue distribution of the apelin-APJ system and their cognate components in RAAS overlap [11]. In physiological experiments, blockade of the RAAS enhanced apelin production in 3T3-L1 adipocytes [28], and APJ-deficient mice showed an increased vasopressor response to Ang II [29]. In study, Gurzu B et al. documented that apelin-13 inhibited Ang II-induced contractions, however, apelin-13 did not modify the isolated rat portal vein tone by itself [30]. All these facts indicated crosstalk between the apelin-APJ system and RAAS, which further supported our results. Furthermore, as RAAS played a crucial role in the pathophysiology of MetS, the reciprocal counter-regulatory effect of the apelin-APJ pathway with RAAS might help to determine the interaction between the apelin-APJ pathway and MetS.

In males, the genetic susceptibility of the apelin-APJ system to glucose homeostasis disorders was revealed. It has previously been reported that DM was associated with plasma apelin concentrations [6–7, 9]. Our work lent support to a contributory role for the apelin-APJ pathway to hyperglycaemia from a genetic standpoint. Moreover, our data demonstrated that the TT genotype of rs10501367 was associated with decreased apelin-36 levels, elevated FPG levels and increased DM risk in males, thus suggesting that the TT genotype of rs10501367 might exert a protective effect on DM through alteration of apelin levels. An association between rs10501367 polymorphism and DM has not been described so far, highlighting a need for functional studies.

Our data indicated that apelin-APJ genetic polymorphisms were correlated with MetS individual components, plasma apelin-36 levels and RAAS. However, the interaction between apelin-APJ system genetic polymorphisms and incident MetS remains to be elucidated. In the single loci analysis, A allele carriers (AA + AG genotype) of rs7119375 conferred a protective effect for MetS in females. Considering that alleles in close proximity on one chromosome might interact with each other, within-gene interactions analysis (haplotype analysis) was conducted, which may enhance the association of individual SNPs in view of that action of single locus on the body physiology is mild [31]. Haplotype analysis demonstrated that the T-A haplotype (alleles in order of rs10501367 and rs7119375) was significantly over-represented in controls for females, which further strengthened the genetic association of rs7119375 with MetS. FastSNP suggested that rs7119375 may modulate transcription-factor binding and subsequently transcriptional activity. Previous studies on rs7119375 had focused on hypertension, which suggested a contributory role of rs7119375 to hypertension in the Chinese population [13, 32], whereas our work was the first to examine rs7119375 in susceptibility to MetS.

Our study also displayed a gender-specific correlation between the apelin-APJ system with FPG, RAAS related parameters and incident MetS. Of note, A allele carriers (AA + GA genotype) of rs7119375 were associated with higher FPG levels than non-carriers for males, however, A allele carriers had a reduced risk of incident MetS in females. A sexually dimorphic pattern for the apelin-APJ system was also documented in other studies [33–35]. The opposite action of apelin-APJ pathway genetic variants on diseases may be attributed to gender-specificity, which resides in sexual chromosome differences and the lifestyle differences.

Several limitations merit adequate consideration. First, the retrospective nature of a case-control study blocked identification of causality. Second, the small sample size made this work underpowered and impaired the robustness of our data. Third, only Han Chinese in Fujian were enrolled and this limited the generalizability of our results. Thus, our work should be viewed as preliminary, and a larger functional study is warranted to replicate our findings, identify causal variants, and explore the molecular mechanisms underlying interactions between the apelin-APJ pathway with MetS in different populations.

In conclusion, these findings offered evidence for the gender-specific involvement of the apelin-APJ system in predisposition to MetS and hyperglycaemia and set the stage for novel diagnostic methods and therapeutic approaches for MetS and hyperglycaemia.

## MATERIALS AND METHODS

### Study population

A total of 1005 subjects who were admitted to the Department of Cardiology, The First Affiliated Hospital of Fujian Medical University from February 2015 to February 2016 were recruited. All study subjects were unrelated Han Chinese, residing in Fuzhou city to the southeast of China. According to previous medical histories and laboratory evaluations, they were categorized into MetS patients (*n* = 448) or controls (*n* = 557). MetS was defined according to the *2007* Chinese guidelines on prevention and treatment of dyslipidaemia in adults [36] when any three of the five alterations were present: visceral obesity (WC > 90 cm in men or > 85 cm in women), elevated TG concentrations (≥ 1.7 mmol/l), decreased HDL-c (< 1.04 mmol/l), elevated arterial blood pressure (BP) (≥ 130/85 mmHg) and dysglycaemia (FPG ≥ 6.1 mmol/l, 2 hours postprandial blood glucose ≥ 7.8 mmol/l or a history of diabetes). Individuals who suffered from secondary hypertension, secondary diabetes, chronic heart failure, heart valve disease, congenital heart disease, cardiomyopathy, chronic kidney disease, pulmonary hypertension or malignant tumour, and individuals who used specific medications including statins, fibrates, angiotensin-converting enzyme inhibitors, angiotensin receptor antagonists and beta blockers were excluded from this study. Study protocols were approved by the ethics committee of The First Affiliated Hospital of Fujian Medical University and written informed consents were obtained from all participants. The study was in line with the guidelines of the Declaration of Helsinki.

### Anthropometric and clinical laboratory measurements

Information on demographic parameters, medical history, medication history, and smoking and drinking history were collected at enrolment by trained observers. Careful physical examination was conducted with all the participants. Body weight, height and WC were measured. BP was taken as an average of three consecutive blood pressure measurements at a 2-minute interval using a calibrated mercury sphygmomanometer with appropriate adult cuff size by experienced examiners in all subjects seated who avoided smoking, coffee, tea and exercise for at least 30 minutes and took a 5-minute rest preceding BP measurement.

Forearm venous blood samples were collected in the morning after a twelve-hour fast and a four-hour supine position. FPG, TG, total cholesterol (TC), HDL-c, low density lipoprotein cholesterol (LDL-c), renin, angiotensin Ι (Ang Ι), aldosterone, epinephrine, norepinephrine, dopamine and other indicators were measured in the clinical laboratory of The First Affiliated Hospital of Fujian Medical University using available kits and auto analyser. ACE2 and Ang II were measured with an enzyme-linked immunosorbent assay (ELISA) kits (R&D Systems, USA) according to the manufacturer’s instructions. Apelin-36, the most widely expressed isoform of apelin [11], were detected using a human apelin-36 ELISA kit (R&D Systems, Inc., Minneapolis, USA) as per the manufacturer’s instructions.

### SNP selection and genotyping

One prior study enrolled 56 MetS subjects randomly for SNP genotyping in the apelin-APJ system, and seven SNPs with a high mutation frequency were chosen, including rs909656, rs5975126, and rs3115757 of *APLN* and rs7119375, rs10501367, rs9943582 and rs11544374 of *APLNR* (Supplementary Table 1).

Genomic DNA was extracted from the forearm venous blood samples with a TIANamp Genomic DNA Kit (Tiangen Biotech (Beijing) Co., Ltd., Beijing, China) based on the protocol of the manufacturer. The concentration and purity of the DNA was determined by spectroscopy. The average DNA concentration was 33.75 ng/μl and the mean OD260/OD280 ratio was 1.82, which satisfied the experimental requirements. The SNPs were genotyped using the polymerase chain reaction-ligase detection reaction (PCR-LDR) method as described previously [37]. Primers and probes of the seven SNPs were designed and synthesized by the Shanghai Generay Biotech Co., Ltd. (Supplementary Tables 2–3). PCR amplification conditions were 94°C (3 min), 35 cycles of 94°C (30 s), 55°C (30 s), 72°C (90 s), and final extension at 72°C (3 min). A multiplex ligation reaction was performed with 30 cycles of 94°C for 30 s and 56°C for 3 min in a 10-ul total reaction volume involving 3 μl of the PCR product, 1μl 106Taq DNA ligase buffer, 0.01 μl of each discriminating probe, and 0.125 μl Taq DNA ligase. 1 μl reaction product mixed with 8 μl loading buffer was denatured at 95°C for 3 min and subsequently chilled rapidly in ice water. Fluorescent products were then differentiated using sequencer.

### Statistical analysis

Data management and statistical analysis was performed with the Statistical Package for Social Sciences (SPSS) 19. 0 software packet. Qualitative data was presented as the mean and standard deviation (mean ± SD). A one-way analysis of variance (ANOVA) and Student’s *t* test were used to compare differences in the anthropometric index and clinical laboratory biomarkers across groups when appropriate. Quantitative data were presented as counts and percentages while a chi-squared test was adopted to compare allele, genotype and haplotype frequencies across groups. Logistic regression analysis was conducted to investigate the effect of alleles and genotypes on MetS after adjustment for confounding factors where ORs with 95% CI were estimated for risk prediction of MetS. Statistical significance was defined as the two-sided *p* value < 0.05.

The statistical analysis was gender-stratified because *APLN* was mapped on the X chromosome. A Hardy-Weinberg equilibrium test was carried out for each SNP with a goodness of fit chi-squared test. Single locus analysis was conducted under a dominant model when appropriate, where the heterozygous genotype plus homozygous variant genotype was compared with the homozygous wild-type genotype. Extent of pairwise linkage disequilibrium between SNPs, which is characterized by |D’| and r^2,^ was calculated utilizing Haploview software version 4.2 [38]. Haplotype frequencies for examined polymorphisms were presented using Haplotype software, while haplotypes with a frequency less than 5% were excluded from further analysis.

## SUPPLEMENTARY MATERIALS FIGURES AND TABLES


