# Supplementary material for: Association study of apelin-APJ system genetic polymorphisms with incident metabolic syndrome in a Chinese population: a case-control study
Source: Oncotarget. 2019 Jun 4;10(38):3807–17. doi: 10.18632/oncotarget.24111 (PMC6557210; doi:10.18632/oncotarget.24111)
Supplement: Supplementary file 1 [file oncotarget-10-3807-s001.pdf]

Association study of apelin-APJ system genetic polymorphisms with incident metabolic syndrome in a Chinese population: a case-control study

SUPPLEMENTARY MATERIALS

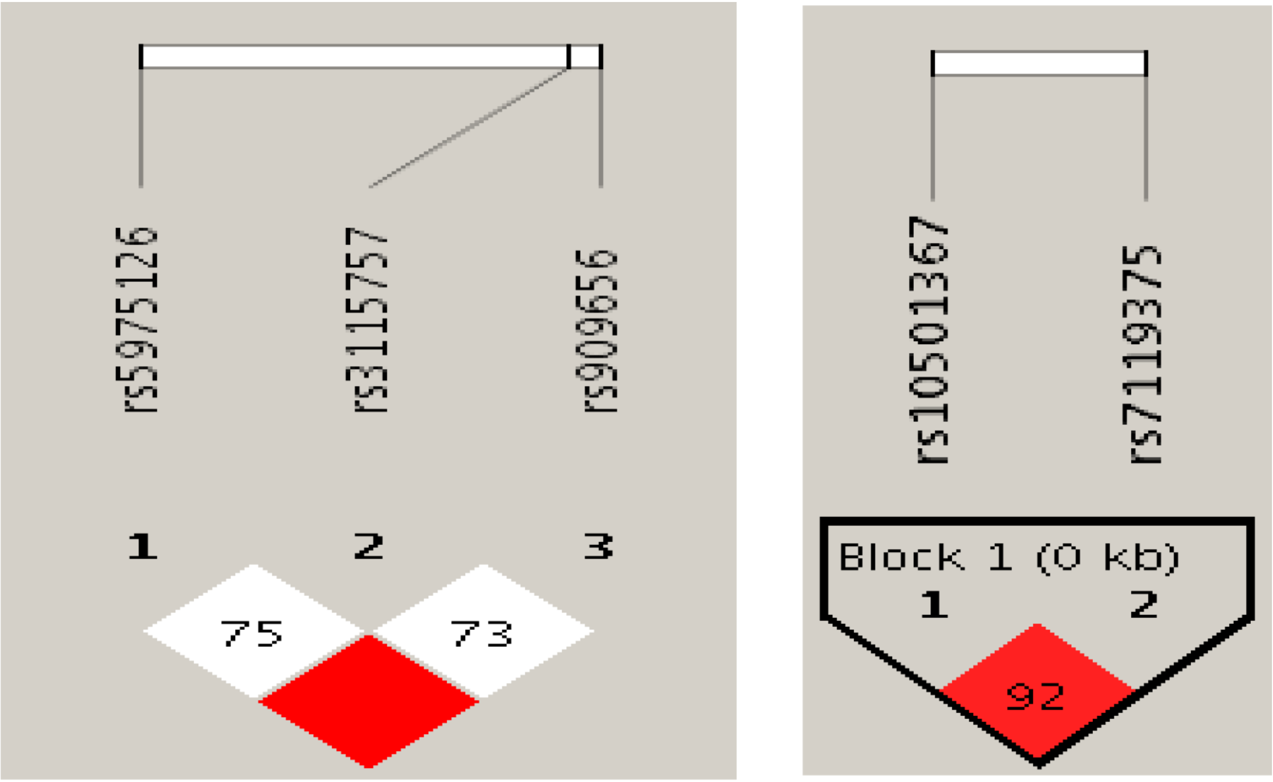

Supplementary Figure 1: Linkage disequilibrium (LD) map for APLN polymorphisms (**A**) and APLNR polymorphisms (**B**). Relative positions for the studied polymorphisms were depicted in the upper panel. Disequilibrium coefficient |D'| expressed as a percentage for every two of the studied polymorphisms was displayed in the cell of the triangle. The r^2 colour scheme of Haploview was applied ($r^2 = 0$ shown in white, $0 < r^2 < 1$ shown in shades of pink, and $r^2 = 1$ shown in red). *APLN*, the apelin gene; *APLNR*, the APJ gene.

Supplementary Table 1: Basic characteristics of the seven analysed SNPs in the apelin-APJ system

SNP ID	Chromosome	Functional Consequence[a]	Allele[b]	Global MAF[c]	MAF in our work	HWE *p* value[d]
APLN						
rs909656	X:129648585	intron variant	[G/T]	2.01%	3.43%	-/ 0.5305
rs5975126	X:129646415	utr variant 3 prime	[G/C]	4.56%	3.08%	-/ 0.5531
rs3115757	X:129648435	Intron variant	[C/G]	41.99%	23.86%	-/ 0.8189
APLNR						
rs7119375	11:57238490	upstream variant 2KB	[G/A]	20.59%	20.15%	0.6218/ 0.4658
rs10501367	11:57238113	upstream variant 2KB	[C/T]	24.62%	23.33%	0.8217/ 0.1704
rs9943582	11:57237593	upstream variant 2KB	[C/T]	30.51%	20.00%	0.3039/ 0.2011
rs11544374	11:57237103	nc transcript variant, utr variant 5 prime	[G/A]	18.53%	16.57%	0.4673/ 0.6710

Quantitative data was presented as counts and percentages. SNP, single nucleotide polymorphism; KB, Kilobase; MAF, minor allele frequency; HWE, Hardy-Weinberg equilibrium; APLN, the apelin gene; APLNR, the APJ gene. a Functional Consequence was obtained from dbSNP. Upstream variant 2KB, upstream of the gene; intron variant, between exons; utr variant 3 prime, within an exon, but not translated, 3' end of the gene; nc transcript variant, transcript variant of a non-coding RNA gene; utr variant 5 prime, within an exon, but not translated, 5' end of the gene. b The alternative bases were shown as the major allele/minor allele. c Data were derived from dbSNP. d p value for a Hardy-Weinberg equilibrium test was expressed as in males/in females. As APLN was located on the X chromosome, Hardy-Weinberg equilibrium test of genetic polymorphisms in APLN was only conducted in females.

Supplementary Table 2: Primers for SNPs

SNP ID	Forward primer	Reverse primer	Amplified fragment length and GC content
rs909656	AACCCCAGGCAGCAGGGTG-GA	TTCTGAAGCAGGTACTT-GAGC	213 bp,GC%:53.99
rs5975126	ACACACACAAAGTTGGGCATC	AGCAAAGGAA-CAGAAGGAAGG	199 bp,GC%:55.78
rs3115757	AACCCCAGGCAGCAGGGTG-GA	TTCTGAAGCAGGTACTT-GAGC	213 bp,GC%:53.99
rs7119375	GAGTGAGTAAGTGGATGGATG	TGAGTCTTTCTCTAGAGA-CCC	211 bp,GC%:45.50
rs10501367	ATGCTTTAGAAGGTTGAGGGC	AAACAGCCATTCACCTAC-GAC	220 bp,GC%:39.55
rs9943582	TGGTTTTGCAAGTCGGCTTTC	CTCAAGCTGGATTG-CAATCTG	212 bp,GC%:58.02
rs11544374	GGCATCTTCAGCCCTTCTTAC	GGATTTCCAGTCTGTG-TACTC	206 bp,GC%:54.85

SNP, single nucleotide polymorphism.

Supplementary Table 3: Probes for SNPs

Locus	Probe sequence
rs909656-TC	TTTTCTTCTTCTCCAAACACGAACATAAC
rs909656-TT	TTTTTTTCTTCTTCTCCAAACACGAACATAAT
rs909656-TR	P-ACAGTACCTGGCATGTCGTAGGTGATTT-FAM
rs5975126-TC	TTTTTTTTTCCGCCCTCCTGTTCTCACCCCCTC
rs5975126-TT	TTTTTTTTTTTTCCGCCCTCCTGTTCTCACCCCCTT
rs5975126-TR	P-CCATCCAATCTAAATGGGACACATTTTTTTT-FAM
rs3115757-TA	TTTTTTTTTTTTTCTTCAGCCCTTCTTACTCTCTGAA
rs3115757-TG	TTTTTTTTTTTTTTTTCTTCAGCCCTTCTTACTCTCTGAG
rs3115757-TR	P-GCTCAAGCCAGAAATTCAGGCTGCTTTTTTTTTT-FAM
rs7119375-TA	AGGGGAATCCATAGATGGAAGAACA
rs7119375-TG	TTTAGGGGAATCCATAGATGGAAGAACG
rs7119375-TR	P-AGTGTAAGAATTACTACGTGGTGAG-HEX-
rs10501367-TC	TTTTCTTCTTCTCCAAACACGAACATAAC
rs10501367-TT	TTTTTTTCTTCTTCTCCAAACACGAACATAAT
rs10501367-TR	P-ACAGTACCTGGCATGTCGTAGGTGATTT-HEX-
rs9943582-TC	TTTTTTTTTCCGCCCTCCTGTTCTCACCCCCTC
rs9943582-TT	TTTTTTTTTTTTCCGCCCTCCTGTTCTCACCCCCTT
rs9943582-TR	P-CCATCCAATCTAAATGGGACACATTTTTTTT-HEX-
rs11544374-TA	TTTTTTTTTTTTTCTTCAGCCCTTCTTACTCTCTGAA
rs11544374-TG	TTTTTTTTTTTTTTTTCTTCAGCCCTTCTTACTCTCTGAG
rs11544374-TR	P-GCTCAAGCCAGAAATTCAGGCTGCTTTTTTTTTT-HEX-

SNP, single nucleotide polymorphism; FAM, 6-carboxy-fluorescein; Hex, hexachloro-fluorescein.

Supplementary Table 4: Ligation reaction product length and labelling

SNP ID	Allele[a]	Product size	Labeling
rs909656	[G/T]	57/G,60/T	FAM
rs5975126	[G/C]	64/C,67/G	FAM
rs3115757	[C/G]	71/C,74/G	FAM
rs7119375	[G/A]	50/A,53/G	HEX
rs10501367	[C/T]	57/C,60/T	HEX
rs9943582	[C/T]	64/C,67/T	HEX
rs11544374	[G/A]	71/A,74/G	HEX

SNP, single nucleotide polymorphism; FAM, 6-carboxy-fluorescein; Hex, hexachloro-fluorescein. [a]The alternative bases are shown as major allele/minor allele.

Supplementary Table 5: Gender-stratified association between apelin-APJ system genetic polymorphisms with MetS individual components, plasma apelin36 levels and RAAS related parameters

SNP	*p* value								
	WC (cm)	SBP (mmHg)	DBP (mmHg)	FPG (mmol/L)	TG (mmol/L)	HDL-c (mmol/L)	apelin36 (ng/l)	Ang II (ng/l)	ACE2 (ng/l)
apelin：rs3761581									
Male (T vs. G)	0.512	0.467	0.805	0.980	0.461	0.285	0.833	0.860	0.623
Female (TT vs. TG vs. GG)	0.131	0.147	0.823	0.328	0.084	0.823	0.353	0.303	0.097
Female (TT+TG vs. GG)	0.476	0.070	0.534	0.179	0.117	0.608	0.227	0.402	0.745
rs909656									
Male (T vs. G)	0.717	0.657	0.911	0.786	0.647	0.365	0.452	0.475	0.905
Female (TT vs. TG vs. GG)	0.330	0.440	0.385	0.918	0.088	0.443	0.373[a]	0.020[a]	0.076[a]
Female (TT+TG vs. GG)	0.379	0.288	0.491	0.705	0.873	0.908	-[a]	-[a]	-[a]
rs5975126									
Male (C vs. G)	0.464	0.671	0.882	0.794	0.904	0.520	0.443	0.493	0.697
Female (CC vs. CG vs. GG)	0.383	0.442	0.492	0.739	0.981	0.810	0.250[a]	0.009[a]	0.049[a]
Female (CC+CG vs. GG)	0.383	0.442	0.492	0.739	0.981	0.810	-[a]	-[a]	-[a]
rs3115757									
Male (C vs. G)	0.611	0.993	0.872	0.797	0.670	0.174	0.619	0.437	0.435
Female (CC vs. CG vs. GG)	0.266	0.521	0.972	0.546	0.927	0.066	0.500	0.071	0.948
Female (CC+CG vs. GG)	0.237	0.470	0.835	0.276	0.973	0.060	0.238	0.021	0.786
APJ：rs7119375									
Male (AA vs. GA vs. GG)	0.202	0.978	0.876	0.006	0.635	0.301	0.116	0.610	0.247
Male (AA+GA vs. GG)	0.276	0.834	0.789	0.012	0.347	0.180	0.848	0.971	0.106
Female (AA vs. GA vs. GG)	0.314	0.203	0.810	0.509	0.749	0.576	0.968	0.831	0.490
Female (AA+GA vs. GG)	0.190	0.140	0.607	0.594	0.485	0.298	0.948	0.654	0.977
rs10501367									
Male (TT vs. CT vs. CC)	0.379	0.459	0.100	0.014	0.955	0.558	0.048	0.796	0.241
Male (TT+CT vs. CC)	0.430	0.216	0.051	0.016	0.837	0.298	0.454	0.717	0.127
Female (TT vs. CT vs. CC)	0.182	0.194	0.543	0.720	0.675	0.276	0.335	0.074	0.082
Female (TT+CT vs. CC)	0.202	0.151	0.275	0.696	0.502	0.117	0.713	0.075	0.530
rs9943582									
Male (TT vs. CT vs. CC)	0.407	0.993	0.292	0.454	0.678	0.426	0.145	0.852	0.599
Male (TT+CT vs. CC)	0.566	0.905	0.574	0.282	0.727	0.251	0.067	0.974	0.413
Female (TT vs. CT vs. CC)	0.670	0.989	0.680	0.649	0.753	0.885	0.999	0.931	0.709
Female (TT+CT vs. CC)	0.389	0.954	0.385	0.505	0.994	0.847	0.981	0.751	0.594
rs11544374									
Male (AA vs. GA vs. GG)	0.285	0.093	0.089	0.067	0.980	0.810	0.138	0.606	0.708
Male (AA+GA vs. GG)	0.304	0.176	0.330	0.092	0.986	0.664	0.504	0.533	0.762
Female (AA vs. GA vs. GG)	0.402	0.257	0.993	0.919	0.842	0.753	0.200	0.172	0.270
Female (AA+GA vs. GG)	0.333	0.257	0.993	0.690	0.648	0.454	0.791	0.260	0.211

SNP, single nucleotide polymorphism; WC, waist circumference; SBP, systolic blood pressure; DBP, diastolic blood pressure; FPG, fasting plasma glucose; TG, triglyceride; HDL-c, high-density lipoprotein cholesterol; Ang II, angiotensin II; ACE2, angiotensin-converting enzyme 2. [a] *p* values were for comparison between heterozygous genotype with homozygous wild-type genotype owing to the missing data for homozygous variant genotype.
